# Supplementary material for: Parental Factors Associated with Child or Adolescent Medication Adherence: A Systematic Review
Source: Healthcare (Basel). 2023 Feb 8;11(4):501. doi: 10.3390/healthcare11040501 (PMC9957533; doi:10.3390/healthcare11040501)
Supplement: Supplementary file 1 [file healthcare-11-00501-s001.zip › healthcare-2096001-supplementary.pdf]

Table S1. Quality assessment of the studies included based on STROBE

Study	1(*a*)	1(*b*)	2	3	4	5	6 (*a*)	6 (*b*)	7	8	9	10	11	12 (*a*)	12 (*b*)	12 (*c*)	12 (*d*)	12 (*e*)	13 (*a*)	13 (*b*)	13 (*c*)	14 (*a*)	14 (*b*)	14 (*c*)	15	16 (*a*)	16 (*b*)	16 (*c*)	17	18	19	20	21	22	Score
Atzori et al. (2009)	1	1	1	1	1	1	1	1	1	1	0	1	1	1	1	0	1	0	1	1	1	1	0	1	1	0	0	0	1	1	1	1	1	0	17
Ayaz et al. (2014)	1	1	1	1	1	1	1	0	1	1	0	1	1	1	0	0	0	0	1	0	0	1	0	1	1	1	0	0	0	1	1	1	0	0	13
Bernstein et al. (2000)	1	1	1	1	1	1	1	0	0	1	0	0	1	1	1	0	0	0	1	0	0	1	0	1	1	0	0	0	0	1	1	1	1	0	12
Burns et al. (2008)	1	1	1	1	1	1	1	1	1	1	1	1	1	1	1	1	1	0	1	0	0	1	0	1	1	0	0	0	0	1	1	1	1	0	16
Bushnell et al. (2018)	1	1	1	1	1	1	1	1	1	1	0	1	1	1	0	0	0	1	0	0	0	1	0	0	1	1	0	1	1	1	1	1	1	1	16
Coletti et al. (2005)	0	1	1	1	1	1	1	-	1	1	0	0	1	1	0	0	0	0	0	0	0	1	0	-	1	0	0	0	0	0	0	1	0	1	10
Dean et al. (2011)	1	1	1	1	1	1	1	-	1	1	0	0	1	0	0	0	0	0	0	0	0	1	0	-	1	0	0	0	0	1	1	1	1	0	11
DelBello et al. (2007)	0	1	1	1	0	1	1	0	1	1	0	0	1	1	0	1	0	0	1	0	0	1	0	1	1	0	0	0	0	1	0	1	1	0	11
Demidovich et al. (2011)	1	1	1	1	1	1	1	1	1	1	0	1	1	1	0	1	0	0	1	1	1	1	1	0	1	0	0	0	0	1	1	1	0	1	16
Drotar et al. (2007)	0	1	1	0	0	1	1	0	1	0	1	0	1	1	1	1	0	0	0	0	0	1	1	0	1	0	1	0	0	1	1	1	1	1	12
Gearing et al. (2009)	0	1	1	1	1	1	1	0	1	0	0	1	1	1	1	1	0	0	1	1	0	1	1	1	1	1	1	0	0	1	1	1	1	0	16
Ghaziuddin et al. (1999)	0	1	1	0	0	1	1	0	1	0	0	0	1	1	1	0	0	0	0	0	0	0	0	1	1	0	0	0	0	1	0	0	0	0	7
Goldstein et al. (2016)	1	1	1	1	1	1	1	0	1	1	0	1	1	1	0	1	0	0	0	0	0	1	0	1	1	0	0	0	0	1	1	1	1	0	13
Harpur et al. (2008)	1	1	1	1	1	1	0	0	1	1	0	0	1	1	0	0	0	0	0	0	0	1	0	0	1	0	0	0	0	0	1	1	0	0	9
Hoza et al. (2000)	0	0	1	0	1	0	0	0	0	0	1	1	0	1	0	0	0	0	0	0	0	1	0	0	1	0	0	0	0	1	1	1	0	0	6
King et al. (1997)	0	1	1	0	1	1	1	0	1	0	0	1	1	1	1	1	0	1	1	1	0	1	0	0	1	0	0	0	0	1	1	1	1	0	13
Moses (2011b)	1	1	1	1	1	1	1	0	1	0	0	0	1	1	1	0	0	0	0	0	0	1	0	0	1	0	1	0	0	1	1	1	1	0	12
Munson et al. (2010)	0	1	1	1	1	1	1	0	1	0	0	0	1	1	1	1	0	0	0	0	0	0	0	0	1	0	0	0	0	1	1	1	1	1	11
Pérez-Garza et al. (2016)	0	1	1	1	1	1	1	0	0	1	0	0	1	1	0	0	0	0	1	0	0	1	0	0	1	0	0	0	0	0	1	1	0	0	9
Pogge et al. (2005)	1	1	1	1	1	1	1	0	1	1	0	1	1	1	0	0	0	0	1	1	0	1	0	1	1	0	1	0	0	1	1	1	1	0	14
Stewart & Baiden (2013)	1	1	1	1	1	1	1	0	0	1	0	0	1	1	0	1	0	0	1	0	0	1	0	0	1	1	0	0	0	0	1	1	1	0	12
Timlin et al. (2014)	0	1	1	1	1	1	1	0	1	1	0	0	1	1	0	0	0	0	1	0	0	1	0	0	1	1	0	0	0	1	1	1	1	0	12
Woldu et al. (2011)	1	1	1	1	1	0	1	0	0	1	0	0	1	1	1	0	0	1	1	0	0	1	0	1	1	0	0	0	1	1	1	1	1	1	14

Note. 6(b) is for cohort & case-control study only; 14(c) is for cohort study only
